# *rac*-Methyl 4-azido-3-hydr­oxy-3-(2-nitro­phen­yl)butanoate

**DOI:** 10.1107/S1600536808043857

**Published:** 2009-01-28

**Authors:** Olivier Vallat, Ana-Maria Buciumas, Reinhard Neier, Helen Stoeckli-Evans

**Affiliations:** a Institute of Chemistry, University of Neuchâtel, Rue Emile-Argand 11, CH-2009 Neuchâtel, Switzerland; b Institute of Physics, University of Neuchâtel, Rue Emile-Argand 11, CH-2009 Neuchâtel, Switzerland

## Abstract

In the title compound, C_11_H_12_N_4_O_5_, the mean plane through the nitro substituent on the benzene ring is inclined to the benzene mean plane by 85.8 (2)°, which avoids steric inter­actions with the *ortho* substituents. The hydr­oxy group is involved in bifurcated hydrogen bonds. The first is an intra­molecular O—H⋯O hydrogen bond, involving the ester carbonyl O atom, which gives rise to the formation of a boat-like hydrogen-bonded chelate ring. The second is an inter­molecular O—H⋯N hydrogen bond involving the first N atom of the azide group of a symmetry-related mol­ecule. In the crystal structure this leads to the formation of a polmer chain extending in the *c*-axis direction.

## Related literature

For literature related to the anti­tumor properties of rhazinilam, see: Bonneau *et al.* (2007 ▶). For literature related to the synthesis and structure–activity relationships of rhazinilam analogues, see: Decor *et al.* (2006 ▶); Baudoin *et al.* (2002 ▶); Ghosez *et al.* (2001 ▶); Rubio & Bornmann (2001 ▶); Dupont *et al.* (2000 ▶, 1999 ▶); Alazard *et al.* (1996 ▶). For details of the Mukaiyama reaction, see: Mukaiyama *et al.* (1974 ▶). For literature related to the synthesis of pyrrolinone precursors, see: Vallat (2004 ▶); Vallat *et al.* (2009 ▶).
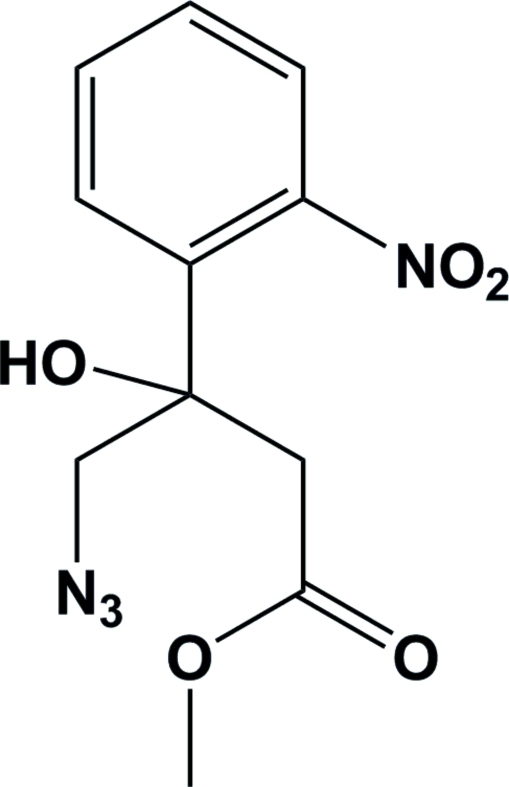


## Experimental

### 

#### Crystal data

C_11_H_12_N_4_O_5_
*M* *_r_* = 280.25
Monoclinic, 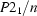

*a* = 9.4772 (11) Å
*b* = 14.0710 (12) Å
*c* = 10.1861 (12) Å
β = 110.496 (13)°
*V* = 1272.4 (2) Å^3^
*Z* = 4
Mo *K*α radiation
μ = 0.12 mm^−1^
*T* = 153 (2) K
0.40 × 0.30 × 0.30 mm

#### Data collection

Stoe IPDS diffractometer
Absorption correction: none
8743 measured reflections
2451 independent reflections
1587 reflections with *I* > 2σ(*I*)
*R*_int_ = 0.074

#### Refinement

*R*[*F* ^2^ > 2σ(*F* ^2^)] = 0.036
*wR*(*F* ^2^) = 0.088
*S* = 0.87
2451 reflections
230 parameters
All H-atom parameters refined
Δρ_max_ = 0.23 e Å^−3^
Δρ_min_ = −0.21 e Å^−3^

### 

Data collection: *EXPOSE* in *IPDS Software* (Stoe & Cie, 2000 ▶); cell refinement: *CELL* in *IPDS Software*; data reduction: *INTEGRATE* in *IPDS Software*; program(s) used to solve structure: *SHELXS97* (Sheldrick, 2008 ▶); program(s) used to refine structure: *SHELXL97* (Sheldrick, 2008 ▶); molecular graphics: *ORTEP-3* (Farrugia, 1997 ▶) and *Mercury* (Macrae *et al.*, 2006 ▶); software used to prepare material for publication: *SHELXL97*.

## Supplementary Material

Crystal structure: contains datablocks I, global. DOI: 10.1107/S1600536808043857/lh2749sup1.cif

Structure factors: contains datablocks I. DOI: 10.1107/S1600536808043857/lh2749Isup2.hkl

Additional supplementary materials:  crystallographic information; 3D view; checkCIF report

## Figures and Tables

**Table 1:** Hydrogen-bond geometry (Å, °)

*D*—H⋯*A*	*D*—H	H⋯*A*	*D*⋯*A*	*D*—H⋯*A*
O3—H3O⋯O4	0.825 (19)	2.30 (2)	2.9439 (16)	135.5 (18)
O3—H3O⋯N2^i^	0.825 (19)	2.27 (2)	2.9193 (18)	135.6 (18)
C10—H10*B*⋯O4^ii^	0.95 (2)	2.557 (19)	3.350 (2)	141.0 (15)
C11—H11*B*⋯O1^iii^	0.95 (3)	2.57 (2)	3.268 (3)	130.9 (16)

Symmetry codes: (i) 
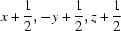
; (ii) 
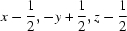
; (iii) 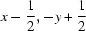, 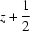.

**Fig. 1.**
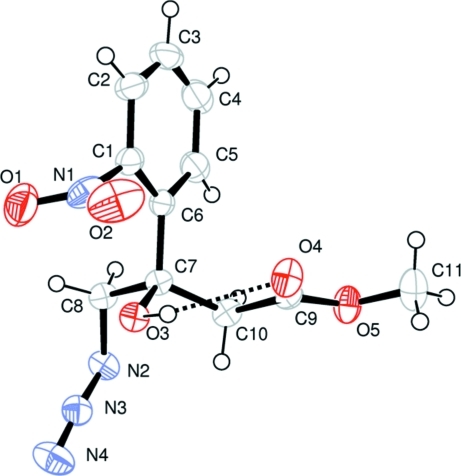
Molecular structure of the title compound, showing the atom labelling scheme and the displacement ellipsoids drawn at the 50% probability level. The intramolecular O—H···O hydrogen bond is shown as a dashed line.

**Fig. 2.**
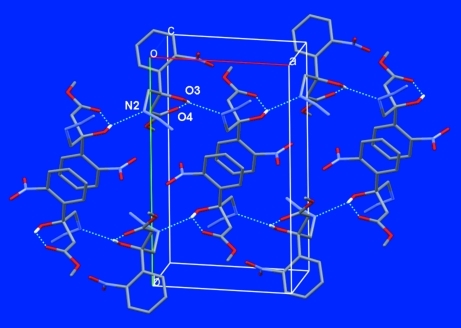
A view along the a axis of the crystal packing of the title compound, showing the intra and intermolecular hydrogen bonds as dashed lines (see Table 1 for details).

## supplementary crystallographic information

### Comment

Rhazinilam, a natural product, has been shown to possess antitumoral properties. It induces *in vitro* spiralization of microtubules [vinblastin effect] and inhibits the disassembly of these microtubules [paclitaxel effect](Bonneau *et al.*, 2007). It has shown significant *in vitro* cytotoxicity towards various cancer cells, but it is not active *in vivo*. Several groups have been interested in synthesizing and studying the structure-activity relationship of rhazinilam analogues (Decor *et al.*, 2006; Baudoin *et al.*, 2002; Ghosez *et al.*, 2001; Rubia & Bornmann, 2001; Dupont *et al.*, 2000; Dupont *et al.*, 1999; Alazard *et al.*, 1996).

In the synthesis of Rhazinilam analogues developed in our group the Mukaiyama reaction, a versatile synthetic tool in organic chemistry, is a key step reaction (Mukaiyama *et al.*, 1974). In one of our retrosynthetic approaches (1-methoxyvinyloxy)trimethysilane was used as a nucleophile, 2-azido-1-(2-nitrophenyl)ethanone as an electrophile and TiCl_4_ as a Lewis acid, to synthesize the title hydroxyester, in high yield. This hydroxyester is a suitable precursor for the formation of the pyrrolinone required for the next step in the synthesis of Rhazinilam analogues (Vallat, 2004; Vallat *et al.*, 2009).

The molecular structure of the title compound is illustrated in Fig. 1. The bond distances and angles are normal. The mean plane through the nitro group is inclined to the benzene mean plane by 85.8 (2)°, so avoiding steric interactions with the *ortho* substituents. The hydroxyl group (O3) is involved in bifurcated hydrogen bonds (Table 1). The first is an intramolecular O—H···O hydrogen bond, involving the ester carbonyl O-atom (O4), and gives rise to the formation of a boat-like hydrogen bonded chelate ring. The second is an intermolecular O—H···N hydrogen bond involving the first N-atom (N2) of the azide group (Table 1). This leads to the formation of a polymer chain extending in the c direction. (Fig. 2). There are also two weak intermolecular C—H···O interactions involving atoms O1 and O4 and the hydrogen atoms of the butanoate moiety (Table 1).

### Experimental

Under an atmosphere of Ar, (1-methoxyvinyloxy)trimethylsilane (1.06 g, 7.3 mmol) was dissolved in dry CH_2_Cl_2_ (15 ml) and the temperature lowered to 243K. 2-Azido-1- (2-nitrophenyl)ethanone (0.5 g, 2.4 mmol) dissolved in dry CH_2_Cl_2_ (6 ml) was added to the reaction mixture dropwise. A solution of TiCl_4_ (0.13 ml, 1.2 mmol), freshly distilled over polyvinylpyridine, in dry CH_2_Cl_2_ (4 ml), was added slowly. The solution became immediately red and then dark red. The reaction mixture was stirred at 243K for 15 min and then at 258K for 30 min. The cold mixture was then poured into an aqueous solution of 2 N NaOH (2.4 ml) and extracted with chloroform. The combined organic layers were washed with brine, dried over MgSO_4_ and concentrated under vacuum. Purification of the residue by flash chromatography (silica gel, CH_2_Cl_2_) followed by crystallization (ether/hexane) gave a white solid (Yield 76%). Colourless plate-like crystals, suitable for X-ray analysis, were obtained by slow evaporation of a solution in ether/hexane (v:v = 1:1)

### Refinement

The H-atoms were located from difference Fourier maps and freely refined: O—H = 0.825 (19) Å, C—H = 0.91 (3) - 1.02 (2) Å.

### Crystal data

**Table:**

C_11_H_12_N_4_O_5_	*F*(000) = 584
*M**_r_* = 280.25	*D*_x_ = 1.463 Mg m^−^^3^
Monoclinic, *P*2_1_/*n*	Mo *K*α radiation, λ = 0.71073 Å
Hall symbol: -P 2yn	Cell parameters from 5300 reflections
*a* = 9.4772 (11) Å	θ = 2.6–25.8°
*b* = 14.0710 (12) Å	µ = 0.12 mm^−^^1^
*c* = 10.1861 (12) Å	*T* = 153 K
β = 110.496 (13)°	Plate, colourless
*V* = 1272.4 (2) Å^3^	0.40 × 0.30 × 0.30 mm
*Z* = 4	

### Data collection

**Table:**

Stoe IPDS diffractometer	1587 reflections with *I* > 2σ(*I*)
Radiation source: fine-focus sealed tube	*R*_int_ = 0.074
graphite	θ_max_ = 25.9°, θ_min_ = 2.5°
φ oscillation scans	*h* = −11→11
8743 measured reflections	*k* = −17→17
2451 independent reflections	*l* = −12→12

### Refinement

**Table:**

Refinement on *F*^2^	Secondary atom site location: difference Fourier map
Least-squares matrix: full	Hydrogen site location: difference Fourier map
*R*[*F*^2^ > 2σ(*F*^2^)] = 0.036	All H-atom parameters refined
*wR*(*F*^2^) = 0.088	*w* = 1/[σ^2^(*F*_o_^2^) + (0.0487*P*)^2^] where *P* = (*F*_o_^2^ + 2*F*_c_^2^)/3
*S* = 0.87	(Δ/σ)_max_ < 0.001
2451 reflections	Δρ_max_ = 0.23 e Å^−^^3^
230 parameters	Δρ_min_ = −0.21 e Å^−^^3^
0 restraints	Extinction correction: *SHELXL97* (Sheldrick, 2008), Fc^*^=kFc[1+0.001xFc^2^λ^3^/sin(2θ)]^-1/4^
Primary atom site location: structure-invariant direct methods	Extinction coefficient: 0.0087 (18)

### Special details

**Table:**

Geometry. Bond distances, angles *etc*. have been calculated using the rounded fractional coordinates. All su's are estimated from the variances of the (full) variance-covariance matrix. The cell e.s.d.'s are taken into account in the estimation of distances, angles and torsion angles
Refinement. Refinement of *F*^2^ against ALL reflections. The weighted *R*-factor *wR* and goodness of fit *S* are based on *F*^2^, conventional *R*-factors *R* are based on *F*, with *F* set to zero for negative *F*^2^. The threshold expression of *F*^2^ > σ(*F*^2^) is used only for calculating *R*-factors(gt) *etc*. and is not relevant to the choice of reflections for refinement. *R*-factors based on *F*^2^ are statistically about twice as large as those based on *F*, and *R*- factors based on ALL data will be even larger.

### Fractional atomic coordinates and isotropic or equivalent isotropic displacement parameters (Å^2^)

**Table:**

	*x*	*y*	*z*	*U*_iso_*/*U*_eq_	
O1	0.32528 (17)	−0.00702 (10)	0.18798 (17)	0.0599 (6)	
O2	0.39794 (16)	0.06178 (11)	0.39169 (15)	0.0653 (5)	
O3	0.20867 (11)	0.19457 (8)	0.17217 (12)	0.0272 (4)	
O4	0.13982 (13)	0.26155 (8)	0.41688 (11)	0.0370 (4)	
O5	−0.07815 (12)	0.34053 (9)	0.35528 (11)	0.0372 (4)	
N1	0.30233 (17)	0.02628 (11)	0.28967 (16)	0.0424 (5)	
N2	−0.03288 (14)	0.22705 (10)	−0.10334 (13)	0.0321 (4)	
N3	0.08103 (17)	0.25861 (10)	−0.12165 (13)	0.0340 (5)	
N4	0.17267 (19)	0.29547 (14)	−0.15016 (17)	0.0535 (6)	
C1	0.1474 (2)	0.01792 (12)	0.29231 (16)	0.0330 (5)	
C2	0.1235 (3)	−0.06299 (14)	0.35997 (18)	0.0472 (7)	
C3	−0.0179 (3)	−0.07913 (17)	0.3639 (2)	0.0574 (9)	
C4	−0.1334 (3)	−0.01579 (16)	0.3016 (2)	0.0524 (8)	
C5	−0.1057 (2)	0.06436 (14)	0.23568 (19)	0.0391 (6)	
C6	0.03622 (18)	0.08457 (11)	0.22890 (15)	0.0277 (5)	
C7	0.05471 (16)	0.17238 (11)	0.14778 (15)	0.0253 (5)	
C8	−0.01067 (19)	0.14495 (13)	−0.00855 (16)	0.0293 (5)	
C9	0.02297 (16)	0.28584 (12)	0.32851 (16)	0.0263 (5)	
C10	−0.02665 (18)	0.25954 (13)	0.17665 (17)	0.0293 (5)	
C11	−0.0434 (3)	0.36959 (19)	0.4988 (2)	0.0489 (8)	
H2	0.210 (2)	−0.1090 (17)	0.404 (2)	0.062 (6)*	
H3	−0.039 (3)	−0.1301 (18)	0.409 (2)	0.071 (7)*	
H3O	0.242 (2)	0.2153 (14)	0.253 (2)	0.043 (6)*	
H4	−0.235 (3)	−0.0267 (16)	0.307 (2)	0.064 (6)*	
H5	−0.183 (2)	0.1080 (15)	0.186 (2)	0.053 (6)*	
H8A	0.0553 (19)	0.0951 (13)	−0.0298 (16)	0.032 (4)*	
H8B	−0.113 (2)	0.1209 (12)	−0.0262 (17)	0.035 (4)*	
H10A	−0.0016 (19)	0.3118 (13)	0.1284 (17)	0.037 (5)*	
H10B	−0.133 (2)	0.2515 (14)	0.1442 (19)	0.047 (5)*	
H11A	−0.035 (3)	0.315 (2)	0.560 (3)	0.084 (8)*	
H11B	−0.128 (3)	0.4060 (17)	0.497 (2)	0.067 (7)*	
H11C	0.044 (3)	0.4065 (17)	0.525 (2)	0.062 (6)*	

### Atomic displacement parameters (Å^2^)

**Table:**

	*U*^11^	*U*^22^	*U*^33^	*U*^12^	*U*^13^	*U*^23^
O1	0.0611 (9)	0.0464 (10)	0.0834 (11)	0.0058 (7)	0.0395 (8)	−0.0098 (8)
O2	0.0494 (8)	0.0595 (10)	0.0596 (9)	0.0016 (7)	−0.0151 (7)	0.0141 (8)
O3	0.0215 (6)	0.0308 (7)	0.0259 (6)	−0.0016 (5)	0.0039 (4)	−0.0006 (5)
O4	0.0298 (6)	0.0426 (8)	0.0307 (6)	0.0080 (5)	0.0006 (5)	−0.0054 (5)
O5	0.0262 (6)	0.0445 (8)	0.0401 (7)	0.0067 (5)	0.0108 (5)	−0.0081 (5)
N1	0.0440 (9)	0.0276 (9)	0.0482 (10)	0.0104 (7)	0.0069 (8)	0.0091 (7)
N2	0.0236 (7)	0.0413 (9)	0.0279 (7)	−0.0005 (6)	0.0048 (5)	0.0044 (6)
N3	0.0352 (8)	0.0377 (9)	0.0257 (7)	0.0053 (7)	0.0063 (6)	0.0049 (6)
N4	0.0431 (10)	0.0645 (12)	0.0571 (10)	−0.0023 (9)	0.0228 (8)	0.0169 (9)
C1	0.0452 (10)	0.0269 (10)	0.0264 (8)	−0.0012 (8)	0.0118 (7)	−0.0025 (7)
C2	0.0829 (15)	0.0269 (11)	0.0336 (10)	−0.0010 (11)	0.0226 (10)	0.0010 (8)
C3	0.109 (2)	0.0334 (13)	0.0435 (12)	−0.0247 (13)	0.0437 (13)	−0.0082 (9)
C4	0.0721 (15)	0.0475 (14)	0.0494 (12)	−0.0280 (12)	0.0360 (11)	−0.0173 (10)
C5	0.0432 (10)	0.0397 (11)	0.0370 (9)	−0.0133 (9)	0.0174 (8)	−0.0097 (9)
C6	0.0341 (9)	0.0255 (9)	0.0229 (7)	−0.0046 (7)	0.0091 (7)	−0.0049 (6)
C7	0.0201 (7)	0.0260 (9)	0.0269 (8)	−0.0015 (6)	0.0045 (6)	0.0003 (6)
C8	0.0272 (9)	0.0297 (10)	0.0272 (8)	−0.0021 (7)	0.0047 (7)	−0.0003 (7)
C9	0.0230 (8)	0.0223 (9)	0.0322 (8)	−0.0018 (7)	0.0080 (7)	0.0002 (7)
C10	0.0226 (8)	0.0299 (10)	0.0304 (9)	0.0017 (7)	0.0032 (7)	0.0012 (7)
C11	0.0456 (12)	0.0569 (15)	0.0476 (12)	0.0028 (11)	0.0207 (10)	−0.0157 (11)

### Geometric parameters (Å, °)

**Table:**

O1—N1	1.224 (2)	C5—C6	1.400 (3)
O2—N1	1.221 (2)	C6—C7	1.530 (2)
O3—C7	1.426 (2)	C7—C10	1.531 (2)
O4—C9	1.206 (2)	C7—C8	1.542 (2)
O5—C9	1.330 (2)	C9—C10	1.497 (2)
O5—C11	1.441 (2)	C2—H2	1.02 (2)
O3—H3O	0.825 (19)	C3—H3	0.91 (2)
N1—C1	1.483 (3)	C4—H4	1.00 (3)
N2—C8	1.473 (2)	C5—H5	0.95 (2)
N2—N3	1.240 (2)	C8—H8A	1.012 (19)
N3—N4	1.133 (2)	C8—H8B	0.983 (19)
C1—C6	1.389 (2)	C10—H10A	0.959 (18)
C1—C2	1.390 (3)	C10—H10B	0.95 (2)
C2—C3	1.374 (4)	C11—H11A	0.98 (3)
C3—C4	1.382 (4)	C11—H11B	0.95 (3)
C4—C5	1.384 (3)	C11—H11C	0.93 (3)
			
C9—O5—C11	116.57 (15)	O4—C9—C10	125.11 (15)
C7—O3—H3O	105.2 (14)	C7—C10—C9	113.54 (14)
O1—N1—O2	125.32 (18)	C1—C2—H2	119.6 (12)
O2—N1—C1	117.49 (15)	C3—C2—H2	121.7 (12)
O1—N1—C1	117.11 (15)	C2—C3—H3	122.3 (18)
N3—N2—C8	116.57 (14)	C4—C3—H3	117.5 (18)
N2—N3—N4	171.07 (18)	C3—C4—H4	120.1 (13)
N1—C1—C6	122.19 (15)	C5—C4—H4	120.3 (13)
C2—C1—C6	123.7 (2)	C4—C5—H5	122.8 (13)
N1—C1—C2	114.10 (18)	C6—C5—H5	114.6 (12)
C1—C2—C3	118.7 (2)	N2—C8—H8A	111.1 (10)
C2—C3—C4	120.3 (2)	N2—C8—H8B	104.2 (10)
C3—C4—C5	119.6 (3)	C7—C8—H8A	109.8 (9)
C4—C5—C6	122.56 (19)	C7—C8—H8B	106.8 (10)
C1—C6—C7	125.77 (16)	H8A—C8—H8B	111.5 (15)
C5—C6—C7	118.97 (15)	C7—C10—H10A	106.5 (11)
C1—C6—C5	115.17 (16)	C7—C10—H10B	112.4 (12)
O3—C7—C6	112.72 (13)	C9—C10—H10A	107.2 (10)
O3—C7—C10	110.15 (13)	C9—C10—H10B	107.4 (11)
C6—C7—C8	105.96 (13)	H10A—C10—H10B	109.6 (16)
O3—C7—C8	104.56 (13)	O5—C11—H11A	111.3 (17)
C8—C7—C10	110.58 (13)	O5—C11—H11B	104.1 (12)
C6—C7—C10	112.49 (13)	O5—C11—H11C	108.2 (13)
N2—C8—C7	113.22 (14)	H11A—C11—H11B	109 (2)
O4—C9—O5	123.38 (14)	H11A—C11—H11C	113 (2)
O5—C9—C10	111.52 (14)	H11B—C11—H11C	111 (2)
			
C11—O5—C9—O4	−1.0 (3)	C4—C5—C6—C1	0.5 (3)
C11—O5—C9—C10	179.32 (17)	C4—C5—C6—C7	177.10 (16)
O1—N1—C1—C2	91.99 (19)	C1—C6—C7—O3	−15.3 (2)
O1—N1—C1—C6	−86.4 (2)	C1—C6—C7—C8	98.52 (18)
O2—N1—C1—C2	−84.7 (2)	C1—C6—C7—C10	−140.54 (16)
O2—N1—C1—C6	96.86 (19)	C5—C6—C7—O3	168.49 (14)
N3—N2—C8—C7	78.29 (18)	C5—C6—C7—C8	−77.74 (18)
N1—C1—C2—C3	−177.75 (16)	C5—C6—C7—C10	43.21 (19)
C6—C1—C2—C3	0.6 (3)	O3—C7—C8—N2	−73.41 (17)
N1—C1—C6—C5	177.43 (15)	C6—C7—C8—N2	167.29 (14)
N1—C1—C6—C7	1.1 (2)	C10—C7—C8—N2	45.12 (19)
C2—C1—C6—C5	−0.8 (2)	O3—C7—C10—C9	−69.34 (17)
C2—C1—C6—C7	−177.19 (15)	C6—C7—C10—C9	57.34 (19)
C1—C2—C3—C4	0.0 (3)	C8—C7—C10—C9	175.59 (14)
C2—C3—C4—C5	−0.3 (3)	O4—C9—C10—C7	19.8 (2)
C3—C4—C5—C6	0.1 (3)	O5—C9—C10—C7	−160.54 (14)

### Hydrogen-bond geometry (Å, °)

**Table:**

*D*—H···*A*	*D*—H	H···*A*	*D*···*A*	*D*—H···*A*
O3—H3O···O4	0.825 (19)	2.30 (2)	2.9439 (16)	135.5 (18)
O3—H3O···N2^i^	0.825 (19)	2.27 (2)	2.9193 (18)	135.6 (18)
C10—H10B···O4^ii^	0.95 (2)	2.557 (19)	3.350 (2)	141.0 (15)
C11—H11B···O1^iii^	0.95 (3)	2.57 (2)	3.268 (3)	130.9 (16)

Symmetry codes: (i) *x*+1/2, −*y*+1/2, *z*+1/2; (ii) *x*−1/2, −*y*+1/2, *z*−1/2; (iii) *x*−1/2, −*y*+1/2, *z*+1/2.
